# Development and Characterisation of Joints with Novel Densified and Wood/Cork Composite Substrates

**DOI:** 10.3390/ma15207163

**Published:** 2022-10-14

**Authors:** Luis M. R. M. Corte-Real, Shahin Jalali, Catarina S. P. Borges, Eduardo A. S. Marques, Ricardo J. C. Carbas, Lucas F. M. da Silva

**Affiliations:** 1 Departamento de Engenharia Mecânica, Faculdade de Engenharia (FEUP), Universidade do Porto, R. Dr. Roberto Frias, 4200-465 Porto, Portugal; 2 Instituto de Ciência e Inovação em Engenharia Mecânica e Engenharia Industrial (INEGI), Campus da FEUP, R. Dr. Roberto Frias 400, 4200-465 Porto, Portugal

**Keywords:** densified wood: biomaterials, adhesive bonding, cork, characterization

## Abstract

The automotive industry, driven by the desire to decrease the environmental impact of vehicles, is permanently seeking to develop lightweight structural components, which lead to lower gas emissions and energy consumption, reducing their carbon footprint. In parallel, adopting innovative, constructive solutions, which dispense non-recyclable and energy-intensive materials, can increase the footprint reduction. Thus, an increase in the use of renewable materials for structural applications, including wood and its by-products, has been observed over the last few decades. Furthermore, composite materials are often joined by using petroleum-based synthetic adhesives, which should be progressively replaced by eco-friendly bio-adhesives. In this study, novel densified wood and wood/cork composites, joined with a bio-adhesive, are proposed and characterised. The densification of the wood aims to enhance the mechanical properties of the natural material, with the purpose of improving the energy absorption of the wood/bio-adhesive joint. To mitigate delamination and the brittle behaviour of wood/cork agglomerates were introduced between the wood substrate and the bio-adhesive. Different configurations of single lap joints (SLJ) were manufactured to study the effect of the overlap length and loading rate on the performance of the joints, both in terms of failure load and energy absorption. Afterward, the joints were numerically simulated. The densification process was successful, although it represents an additional challenge in terms of surface flatness, because the bio-adhesive requires zero bondline thickness. The increase of the overlap had a positive impact on the energy absorption of the joint, and the addition of cork resulted in a more consistent failure mode and higher strain to failure. The numerical models developed had a good correlation with the experimental results.

## 1. Introduction

Adhesive bonding technology is an increasingly attractive alternative to conventional mechanical joints in diverse industries, such as those operating in the automotive, aeronautical, aerospace, and electronics sectors. In comparison to typical joining solutions, such as fastening and riveting, adhesive joints present several important advantages. One of these advantages is the continuous nature of an adhesive joint, which promotes a more uniform stress distribution along the bonded area, improving load transmission and fatigue resistance. Well-designed adhesive joints lead to important reductions in the weight and cost of the final structure. Other advantages include the ability of this technology to join dissimilar materials and the improved flexibility it brings to the joint design [1].

Wood is a highly anisotropic natural material that represents a sustainable option for multiple structural applications, even in cases where steel, aluminium, and concrete have historically been employed. The usage of wood has recently gained a significant importance, driven by the fact that this material has a low carbon impact and is an entirely renewable resource, especially if considering forestry and harvesting practices which ensure the long-term health and diversity of forests [2]. For this reason, significant interest lies in the utilization of timber derived from fast-growing species. However, these woods normally exhibit low density and poor mechanical properties, which strongly limits their use in advanced engineering structures and other applications [3,4]. Nevertheless, an eventual increase of these materials’ density has the potential to convert them into high-value and high-performance products, acting as a substitute to harder species or even some conventional metallic alloys in quite demanding applications [5].

Wood joints are crucial for many material applications and are commonly carried out by using adhesives. The shear strength of a joint is a widely used indicator to evaluate the quality of glued wood products. The factors which affect it include the physical and chemical properties of the adhesive, the physical and structural properties of the wood substrates, and the parameters of the process [6,7]. An indicator which has commonly been employed to measure the quality of a wood adhesive joint is wood failure percentage (WFP), which defines the percentage of area in the joint where the failure occurred in the wood. The higher this value is, the better the quality of the bondline and, ultimately, the strength of the joint is the only function of the wood [8]. Due to the cellular nature of wood, absorption of the adhesive by the substrate often occurs, and its level depends largely on the density of the wood and, consequently, on its porosity. Follrich et al. (2008) [9] studied the effect of the density of spruce on the quality of end joints and the results showed that the bond strength increased as the density increased. However, in a following study, Follrich et al. (2010) [10] conducted further research on balsa wood end joints considering differing densities. Here, the increase in density promoted a rapid decrease in WFP and a slight increase in bond strength.

Therefore, different treatments were developed so that wood, a low-density and low-strength material, had an increase in its mechanical properties. These treatments led to an extension in the possible range of wood applications, including as substrates in structural adhesive joints. Several technological processes have been proposed in recent years to increase wood density, most of which involve thermo-hydromechanical (THM) treatments [5]. The reason for this relies on the viscoelastic nature of wood, which make its mechanical properties depend on time, temperature, and moisture. Like other amorphous polymers, the lignin and hemicellulose present in wood also have a glass transition temperature (T_g_) that marks the transition between a stiff and brittle behaviour (below T_g_) and a rubbery and compliant one (above T_g_). Such behaviour is promoted by polymer mobility and a consequent rearrangement of the molecules enabled by temperatures above the T_g_ of these polymers, which allows for large deformation under compression to occur through buckling of the cell walls, instead of a fracture process [11,12,13]. A wood’s T_g_ is also greatly influenced by moisture, as an increase in the moisture content of wood results in a decrease of the T_g_. It acts as a plasticiser, reducing the secondary bonding between the polymer chains and allowing the polymer molecules to move around more easily [12].

A major drawback associated with the densification process has to do with the resultant moisture sensitivity, which can lead to irreversible swelling—a phenomena known as “set-recovery” [13]. The reasons behind this are attributed to the release of elastic energy stored in the cellulose macromolecules and the unbroken and, therefore, not reformed covalent and hydrogen bonds between the polymers [12,13,14]. Sadatnezhad et al. (2017) [15] focused on increasing the density in the region beneath the surface of poplar wood by applying a THM treatment. The set-recovery after three wet-drying cycles was 44%. Laine et al. (2016) [16] experienced a set-recovery of 60% after water-soaking densified Scots pine samples. However, this was was almost eliminated by the application of a thermal modification treatment after the compression. Schwarzkopf reduced the set-recovery on densified poplar, spruce, and beech samples by impregnating a phenol resin into the cell lumens prior to the THM treatment [14]. Chemical pre-treatments have also been successfully employed to solve this issue and generally consist of the utilization of an alkaline solution to enable reactions that promote the partial removal of lignin and hemicellulose [4,17,18]. The end result is a more flexible and formable material with increased porosity which retains its fibrous cell wall structure and is easily compressed without defects [19,20]. Song et al. (2018) [4] proposed a boiling process in a solution of NaOH and Na${}_{2}$SO${}_{3}$ followed by hot pressing of several species of softwood, obtaining a specific strength higher than most of the structural alloys. Novel et al. (2020) [18] performed a similar chemical treatment before incorporating nanoparticles of silicon carbide or graphene oxide and proceeding to the pressing stage. The addition of the nanoparticles resulted in a reduced water uptake by the wood. Jiangtao et al. (2020) [17] followed a similar procedure and obtained an average compression ratio of 80% in the radial direction of the wood. However, the effect of the use of densified wood on the behaviour of adhesive joints is not yet clearly established.

The adhesive used in this study is a polyurethane-based bio-adhesive. Polyurethane adhesives have been seen to lead to good joint strength; however, they can be sensitive to the moisture content of the wood and to temperature, showing worse bonding quality as the temperature increases and wood humidity decreases [21,22]. Vasiliki et al. (2017) [23] analysed the bondability of a polyurethane adhesive and a polyvinyl acetatea adhesive by using black locust wood and beech wood substrates and concluded that pressure applied to the joint, regardless of adhesive and substrate, negatively influences shear bond strength, which was attrituted to the relation between the structure of the wood and its density and the characteristics of the adhesive, such as its viscosity and density. Additionally, it was noted that many factors can have an influence on the bondability of polyurethane adhesives and must be considered, such as wettability of the substrate surface and the ratio between the stiffness of the adhesive and substrate.

Structural adhesive joints can also benefit from the inclusion of biomaterials within the adhesive layer. To increase the toughness of an adhesively bonded joint, the inclusion of cork particles can also be considered [24]. Akhavan–Safar et al. (2018) [25] studied the effects of natural cork particles on the microfailure mechanisms and static strength of an epoxy adhesive and obtained a higher energy absorption and increased toughness for bulk specimens containing 1% in volume of cork particles. The results also showed that the optimum amount of cork may vary depending on the loading conditions. Da Silva et al. (2021) [26] focused on the incorporation of cork microparticles on an epoxy adhesive as a tool to promote cohesive failure in single-lap joints. The results showed that for some certain conditions the performance of the joint was improved, although the failure mechanisms in these cases was adhesive, between the adhesive and substrate. Therefore, the inclusion of cork to the wood composite, is believed to improve the energy absorption of the joint, making it more suitable for impact conditions, but the behaviour is not yet known with certainty.

In this work, adhesive joints that are aimed to represent a more sustainable alternative to the traditional composite/petroleum-based joints were tested. For that, pine wood was used as a substrate and bonded by using the bio-adhesive. To improve the mechanical properties of the pine wood substrate, a novel method to densify the material was used. Afterward, cork was added to the wood substrate to improve overall energy absorption of the joint. Different overlap lengths were analysed.

## 2. Experimental Details

### 2.1. Materials

#### 2.1.1. Wood

**Figure 1:**
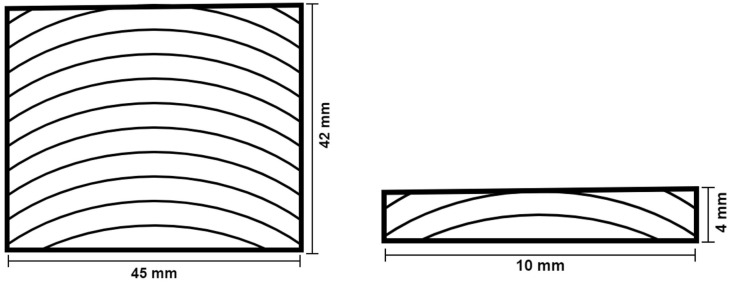
Illustration of the supplied cross-section dimensions of pine, highlighting the preferable ring orientation.

In this work, maritime pine timber (*Pinus pinaster*) was the main material utilized, because it is an easy to work with, cost-effective, strong, and durable wood. It was supplied in two different cross-sections. The beams which had a larger cross-section were used for the densification process and other support procedures, whereas beams with a smaller cross-section were employed in the manufacturing of the substrates for single-lap joints (SLJ). It must be stated that having symmetry of the wood rings in relation to a centered vertical axis was not always possible; however, the preferable orientation of the rings is shown in Figure 1. The density of the supplied timber varied in the range between 0.564 g/cm^3^ and 0.670 g/cm^3^.

The nine elastic constants and strength properties on the longitudinal (L), radial (R), and tangential (T) directions can be determined experimentally. Moura and Dourado [27] have published these properties for pine wood, summarized in Table 1 and Table 2, respectively. In the tables, $E_{i}$ are the Young’s moduli, $G_{i}$ are the shear moduli, $\nu_{i}$ the Poisson’s ratios, and $\sigma_{i}$ the strength.

#### 2.1.2. Bio-Adhesive

**Figure 2:**
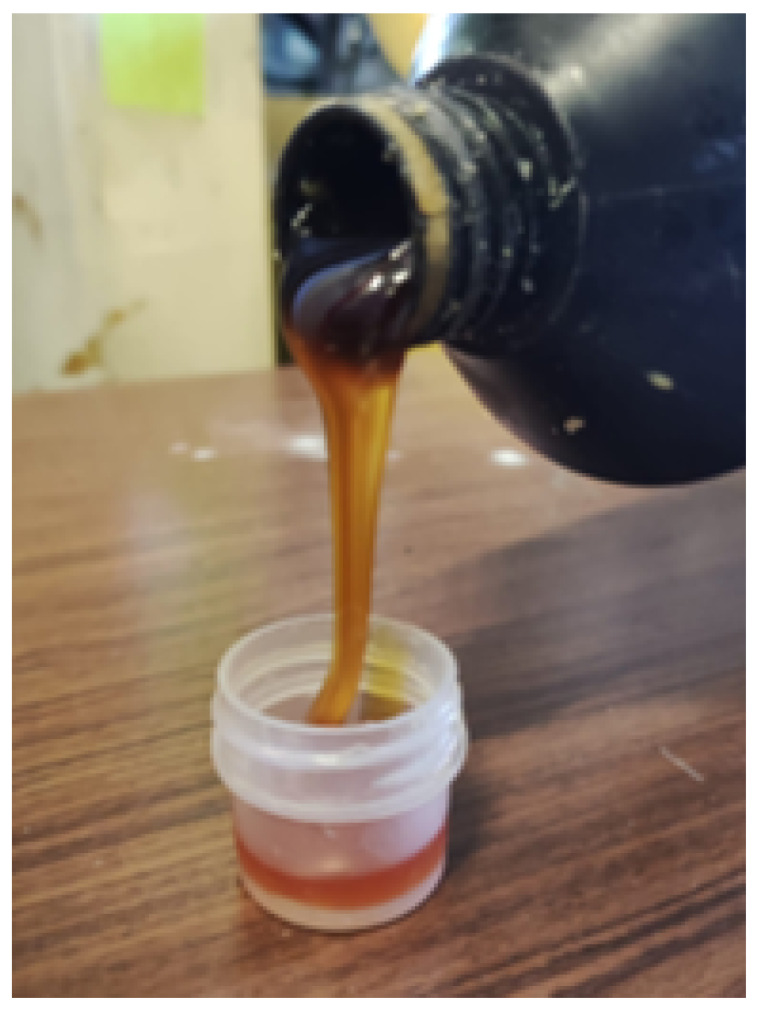
Bio-adhesive supplied for this work.

The adhesive supplied (Figure 2) was developed by Professor João Bordado, from Instituto Superior Técnico. It is a polyurethane-based bio-adhesive, with 70% of its content derived from natural sources. It is known to provide good adhesion to both wood and cork. This bio-adhesive is a prototype material and is not yet commercialized.

Obtaining the properties of the adhesive was found to be a complex task, due to the fact it only cures in porous wood substrates and must be employed in zero thickness layers. Regarding mechanical characterisation, a minimum shear strength value of 12.40 MPa was obtained with testing of a modified thick adherend shear test (TAST) specimen. $G_{Ic}$ and $G_{IIc}$ values of 0.16 N/mm and were obtained from previous testing of modified double-cantilever beam (DCB) and end-notched flexure (ENF) specimens.

### 2.2. Densification Process

#### 2.2.1. Chemical Treatment

Each of the wood blocks, with the the cross-section dimensions already mentioned before and an average length of 240 mm, was subjected to a chemical treatment prior to the compression stage. The chemical bath was based on the work of Song et al. (2018) [4] and consisted of an aqueous solution of 2.5 M of NaOH and 0.4 M of Na_2_SO_3_. Thus, 250 g of NaOH and 125 g of Na_2_SO_3_ were carefully dissolved in 2.5 L of deionized water inside a metallic container. The wood block was then submerged on the solution, and the container was kept in an oven at boiling temperature (100 ${}^{\circ }$C) for 7 h. Finally, the material was kept in two baths in boiling deionized water (1 h), allowing us to remove any chemicals that remained in the wood.

#### 2.2.2. Thermo-Mechanical Treatment

**Figure 3:**
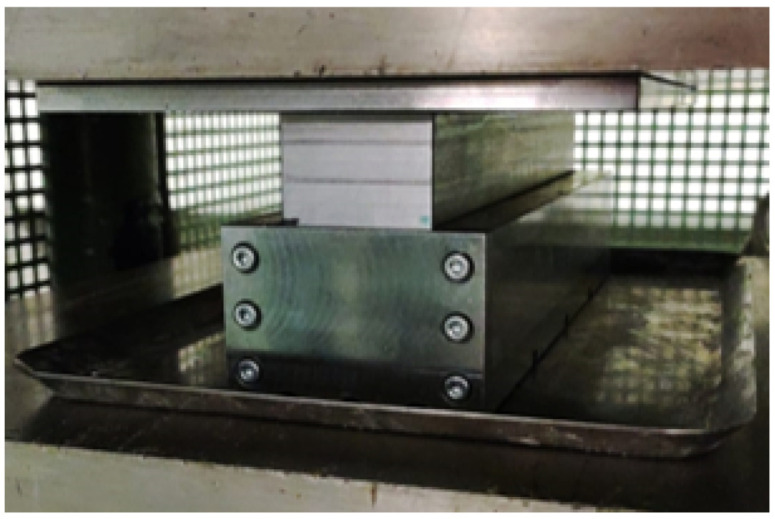
Mould utilised for the densification process.

**Figure 4:**
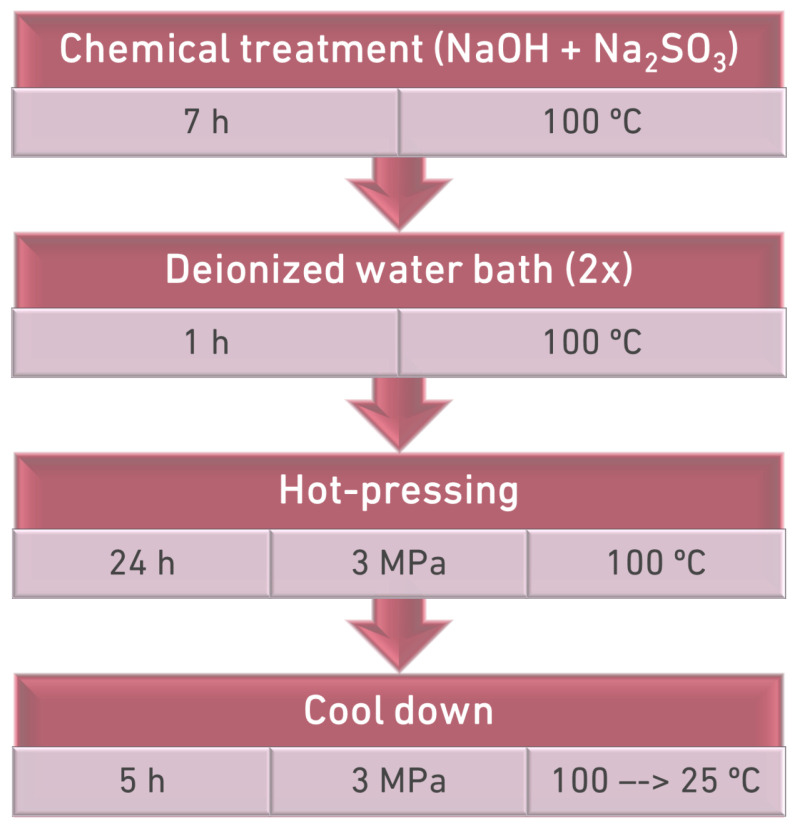
Steps of the densification process.

Immediately after removal from the deionized water bath, the wood block was treated in a hot press for 24 h at 100 ${}^{\circ }$C and 3 MPa. Initially, the timber blocks were placed directly on the hot press with no lateral restriction. However, this resulted in excessive lateral displacement of the compressed wood, creating cracks and other defects. To avoid these issues in the final product, a steel mould was designed and employed in the compression stage to restrain the movement in the lateral direction (Figure 3). Following the compression stage, the blocks were cooled down to room temperature for 5 h, before being removed from the hot press. The steps of the global process, including chemical pre-treatment, are specified in Figure 4.

### 2.3. Joint Manufacturing

**Figure 5:**
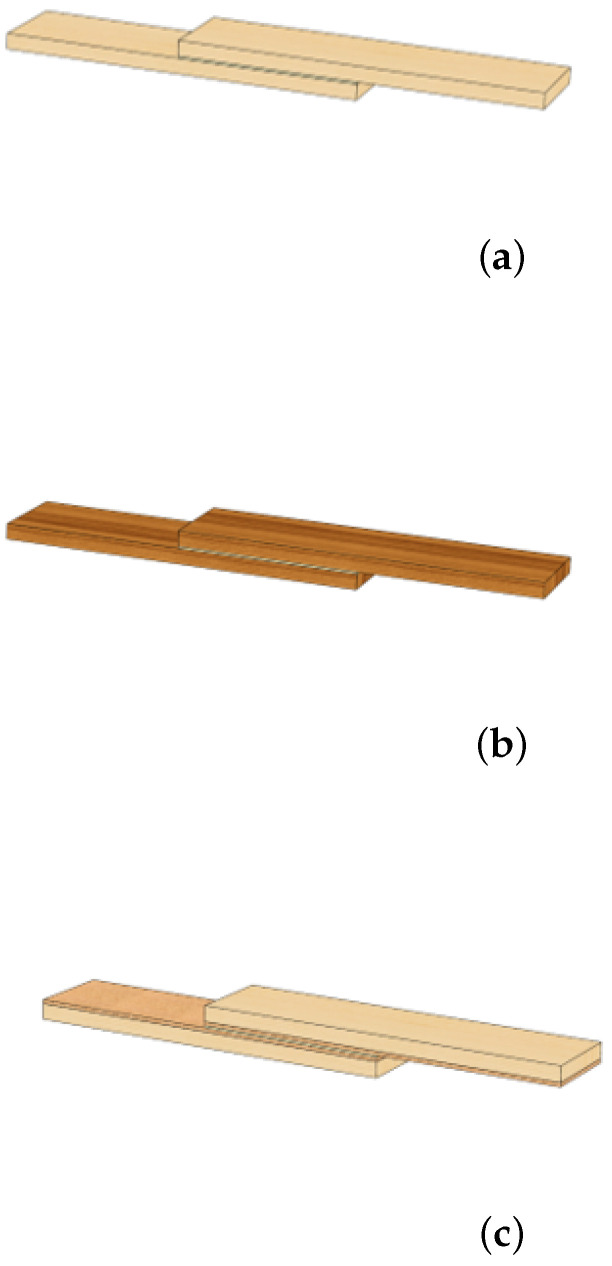
Illustration of the SLJs tested: (**a**) W. (**b**) DW. (**c**) WC.

Three different configurations of SLJs were considered and manufactured:

− SLJs with timber adherends (W)—Figure 5a;
− SLJs with densified timber adherends (DW)—Figure 5b; and
− SLJs where the adherend consisted of timber coated with a cork agglomerate (WC)—Figure 5c.

**Figure 6:**
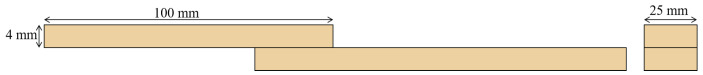
Dimensions of the SLJs tested.

For all cases, the joints were manufactured based on ASTM D1002 [28] and ISO 4587:1995 [29] with overlap lengths of 25 and 50 mm. The substrate dimensions were 100 × 25 × 4 mm, Figure 6. The joints were cured over a period of 8 h at 50 ${}^{\circ }$C with pressure applied through the use of manual clamps. For the cork-coated specimens, the cycle lasted double the time, because the process of bonding the coating to the wood substrate required the same curing cycle used in joint preparation. The thickness of the adhesive layer was not controlled, because the bio-adhesive in use can only be successfully cured in a zero thickness condition due to its very low viscosity. After the joints were manufactured, they were kept in controlled laboratory conditions of temperature and humidity until testing.

### 2.4. Testing Conditions

The SLJs manufactured for this work were tested via the application of a tensile load on the substrates and were conducted in three different test machines, according to the testing rate defined: quasi-static (Q-S), high cross-head rate (HCR), and impact. Because the target application of these adhesive joints are vehicles, different testing speeds need to be tested to understand the behaviour of the material at low and higher velocities, such as in the case of a collision. The testing campaign is defined in Table 3. It is crucial to note that cross-head rate was considered as the key testing parameter in these testing procedures due to the impossibility of calculating the shear strain rate in the adhesive layer, because the zero layer thickness of the layer precludes this calculation. At least five specimens were tested for each condition. The tests were conducted in laboratory conditions of temperature and humidity.

For the quasi-static tests, the universal test machine INSTRON^®^ 3367 with a load cell capacity of 30 kN was used and both the load and displacement were recorded by the machine at a frequency of 10 s${}^{-1}$.

The higher loading rates were attained in the servohydraulic test machine INSTRON^®^ 8801 with a load cell capacity of 100 kN and both load and displacement were recorded by the machine at a frequency of 2000 s${}^{-1}$.

As for the impact tests, a drop-weight machine was used. A mass of 50 kg was dropped from a height of 51 mm to achieve the desired velocity. The acquisition system recorded load and time at a frequency of 50,000 s${}^{-1}$, although, to measure displacement, digital image correlation (DIC) was required. Thus, part of the bottom clamp was speckled with paint and its movement tracked with a high-speed camera filming at 10,000 frames per second.

## 3. Experimental Results and Discussion

### 3.1. Densification Process

**Figure 7:**
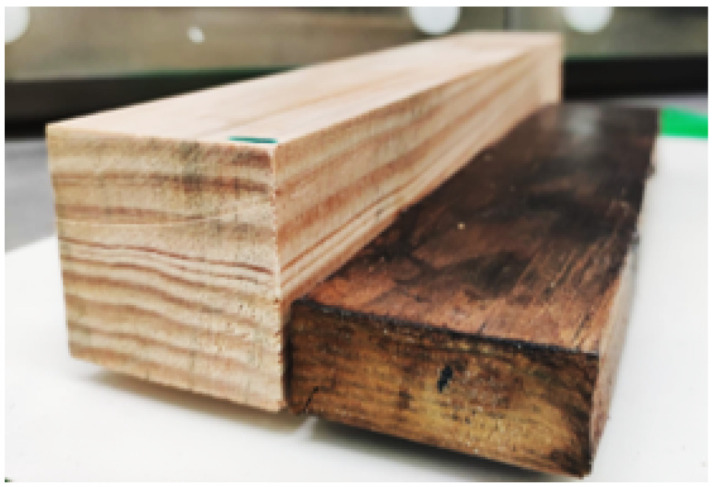
Visual comparison between a natural block and a densified block (with mould).

In this section, results from the densification process applied to the timber blocks and from the joints tested are presented. After each densification process was completed, the densified timber blocks were removed from the mould, weighted and measured to assess its final density. In Table 4, the resulting density of several blocks is presented, where the effect of the implementation of the mould in the process is clearly noticeable (Figure 7).

From Table 4, it can be seen that the densification process adopted leads to an increase in density of 62 ± 5%, which is already a significant result. However, to further promote the densification, a mould was developed and, with the mould, an increase of 123 ± 16% was achieved. This shows that the densification process used is effective; however, it can still be significantly improved through the use of the mould.

**Figure 8:**
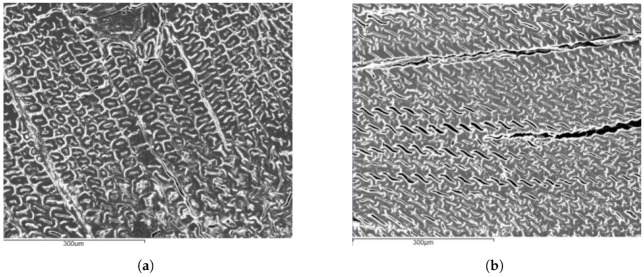
SEM images of wood. (**a**) Natural. (**b**) Densified (without mould).

In Figure 8, SEM images of a cross-section perpendicular to the longitudinal direction are presented for both natural pine wood (Figure 8a) and densified wood, although without mould (Figure 8b). Collapsing of the cell wall is observable, but some gaps are still present, which was believed to have decreased with the introduction of the mould to the process.

The process was found to be greatly improved by the introduction of the mould, resulting in a material which had double the final density of the original wood. It also enabled us to reduce the dispersion of the density increase level, stabilising its value around the 110–120% region. Furthermore, the final product presented almost no visible external defects.

### 3.2. Effect of the Overlap Length on Failure Load and Energy Absorbed

Tests carried out under a quasi-static rate were used to understand the influence of the overlap length in the behaviour of the SLJs. These results are presented and discussed in the following sections.

#### 3.2.1. Wood–Wood Joints (W)

**Figure 9:**
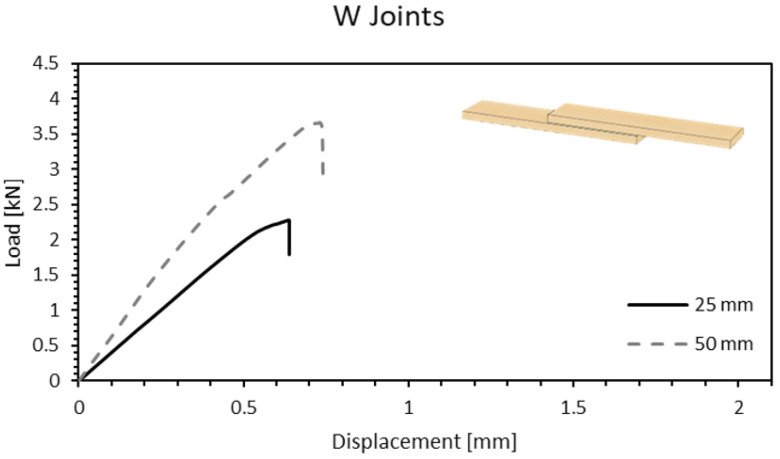
Load-displacement curve of a W joint for 25- and 50-mm overlap length.

For the most simple configuration, a W joint, it can be observed in Figure 9 that it benefits from an increase in overlap length, presenting higher stiffness and almost double the failure load for a 50-mm overlap. The average values obtained for the lap shear strength (LSS) were 3.88 MPa for the 25 mm overlap and 2.99 MPa for the increased overlap length. To understand this variation, an analysis of the failure modes should be conducted.

**Figure 10:**
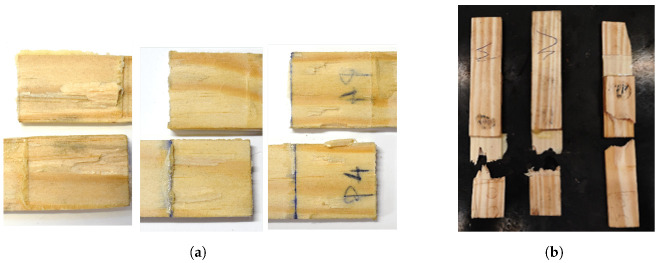
Failure mechanisms in W joints. (**a**) A 25-mm overlap. (**b**) A 50-mm overlap.

From the analysis of the typical failure modes witnessed on experiments, observed in Figure 10a, one could conclude that the delamination of adherends was the main cause of failure. As the overlap length was increased, the joint began to fail predominantly on the timber substrates and the bondline, in most cases, remained intact (Figure 10b). Delamination was somewhat expected to happen due to the relatively low strength of timber in the transverse directions—a process similar to what occurs in bonded composite materials. In this case, strength in the radial direction was not enough to sustain the peel stresses generated during the test. With a larger overlap length, however, the increase in resistant area was significant enough to shift the critical point toward the timber itself. The failure stress was computed by having the timber cross-section in consideration and the value obtained was, on average, 22.5 MPa. This value was still found to be quite far from the average tensile strength values reported in the longitudinal direction for pine wood (97.5 MPa), thus confirming the prominent contribution of other loading mechanisms on the joint besides pure shear.

#### 3.2.2. Densified Wood–Densified Wood Joints (DW)

**Figure 11:**
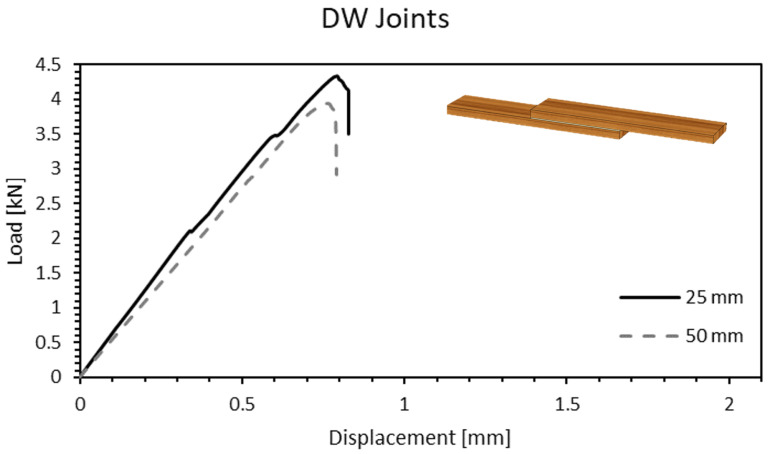
Load-displacement curve of a DW joint for 25- and 50-mm overlap length.

After the wood densification was carried out, the adherends were replaced by densified timber. In this case, larger failure loads were to be expected, because the enhancement of mechanical properties is generally proportional to the increase in density of the material. Follrich et al. (2008) [9] hypothesized that the bonding performance of a spruce end joint would diminish with an increase in density due to reduced penetration of the adhesive in the cell cavities. However, for this adhesive/substrate combination, the experimental results proved the opposite. In fact, the results showed a failure load 87% higher on average than in natural timber (Figure 11). Despite these encouraging results, the increment in overlap length led to an average failure load slightly lower than the one observed for the shorter overlap. The reason might derive from the difficulty in ensuring a zero thickness condition on densified samples, where a flat surface was harder to obtain. Furthermore, larger overlaps also increase the chances of having areas where a zero thickness condition cannot be properly achieved.

**Figure 12:**
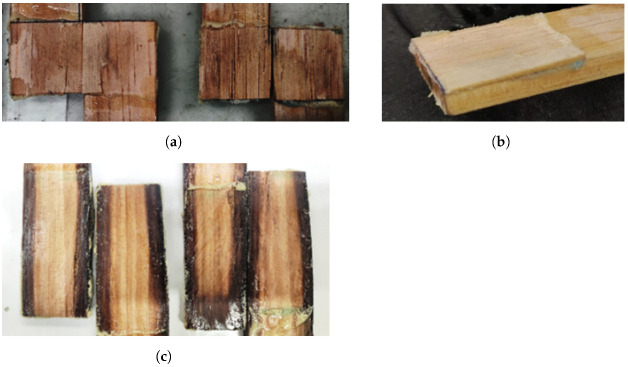
Failure mechanisms in DW joints. (**a**) A 25-mm overlap. (**b**) Detail of 25-mm overlap. (**c**) A 50-mm overlap.

The failure modes presented in Figure 12a suggest that, despite the density improvement, delamination still occurs in varying degrees of intensity, being the specimen observed in Figure 12b an example of an extreme scenario where the wood failure percentage was maximum. The 50-mm overlap joints observed in Figure 12c reflect the aforementioned hypothesis of improper bonding between the substrates. A lack of any delamination signs combined with a low failure load was probably a result of failure of the bio-adhesive, meaning that zero thickness was not properly achieved in the majority of these samples.

#### 3.2.3. Wood/Cork—Cork/Wood Joints (WC)

**Figure 13:**
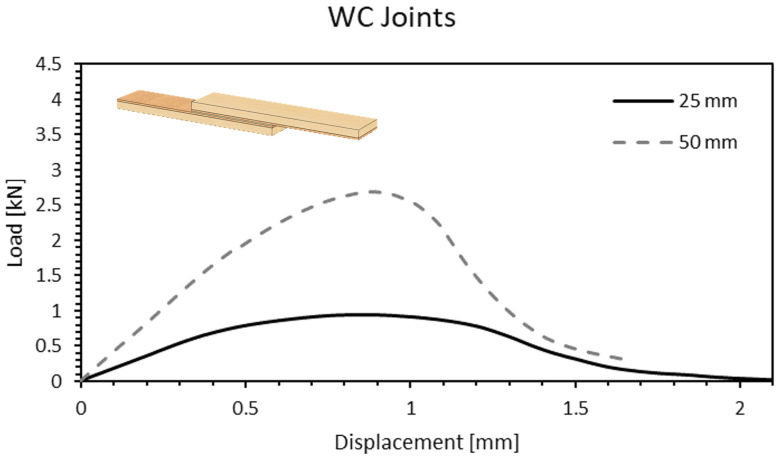
Load-displacement curve of a WC joint for 25- and 50-mm overlap length.

In Figure 13, representative curves for the WC joints are displayed. A clear difference on the behaviour of the joint is noticeable, where a brittle curve previously observed in Figure 9 and Figure 11 is replaced by a ductile one which presented a much more controlled failure. The downside resides in the value of the failure load which, referring to a 25-mm overlap, shows a decrease of 58% and 77% when compared to the W and DW joints, respectively. Nonetheless, due to the shape of its curve resulting in a larger area under it, the WC joint shows an improvement of the amount of energy absorbed during the test. Furthermore, an increase of the overlap length was found to have the greatest impact on this type of joint, improving its failure load by 200%, on average, and increasing the amount of energy absorbed by 150%.

**Figure 14:**
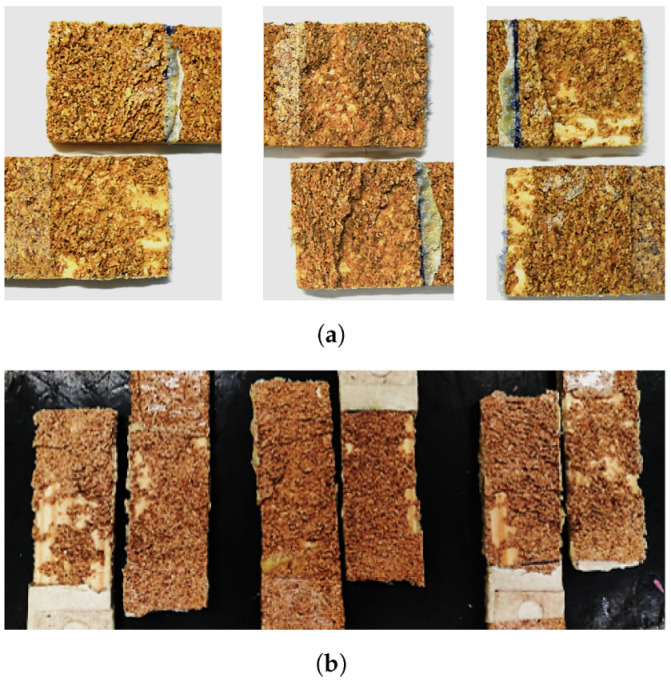
Failure mechanisms in WC joints. (**a**) A 25-mm overlap. (**b**) A 50-mm overlap.

Examination of the joints following the testing procedure demonstrated a failure dictated by a combination of the adhesion between cork particles and adhesion between cork and timber. The failure mechanism was found to be similar for all the tests performed and generally independent of the overlap length (Figure 14), almost certainly owing to low strength values of the cork agglomerates.

#### 3.2.4. Comparison between Different Substrates and Different Overlap Lengths

**Figure 15:**
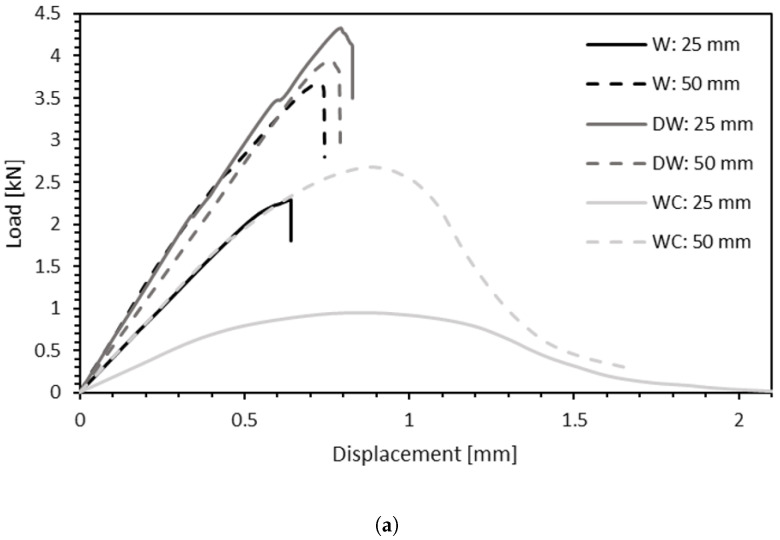

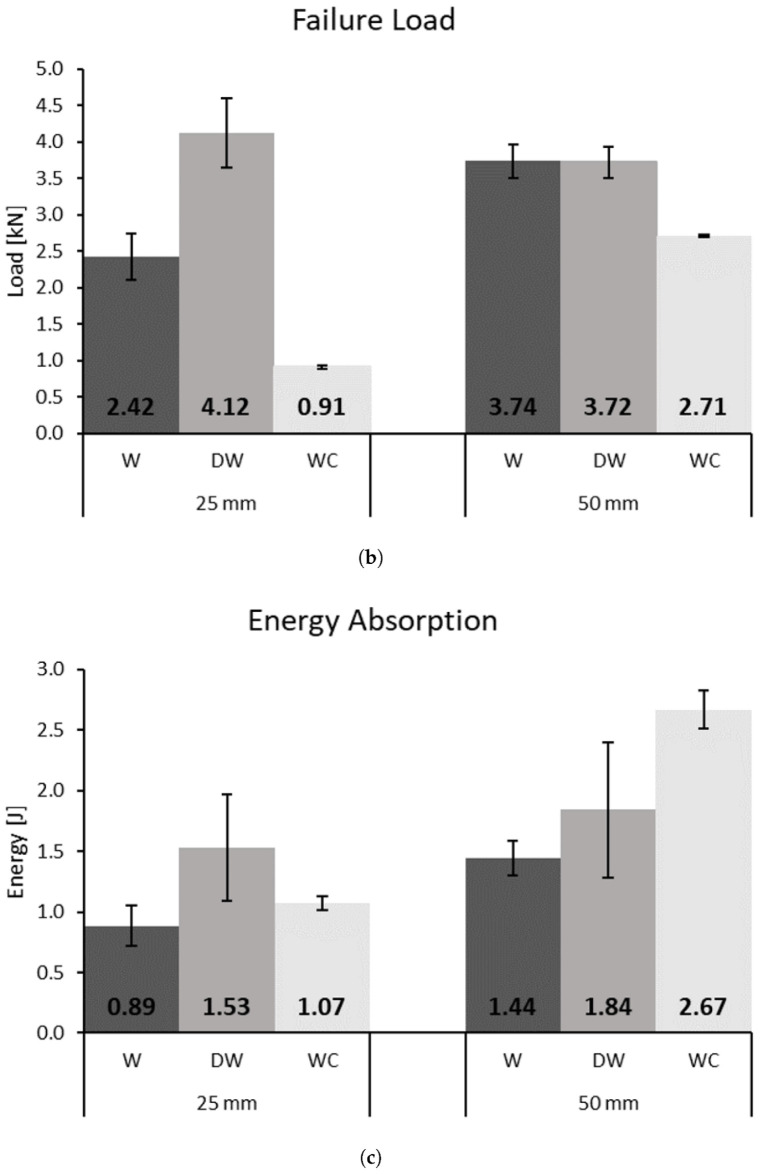
Summary of results. (**a**) Representative curves for all test conditions. (**b**) Summary of failure loads for each joint configuration and overlap length. (**c**) Summary of energy absorption values for each joint configuration and overlap length.

In Figure 15a, both the effect of the densification and the introduction of cork are distinguishable and comparable to natural pine wood. Figure 15b,c provide a comparison between the parameters evaluated for all conditions tested.

For both overlap lengths, it can be seen that the DW joint had a higher energy absorption, which is compatible with the strength increase of the densified wood reported by Song et al. (2018) [4], but the potential of the densified wood was not fully taken advantage of for 50-mm overlap because the failure path shifts from the substrate to the adhesive layer. When moving from an overlap of 25 mm to 50 mm, the failure load usually increased due to an increase of the resistant area, the exception being the DW joints, because the failure was not on the substrate for the higher overlap. Regarding the WC samples, it can be seen that, although the failure load decreases, the energy absorption of the joint was higher, particularly for an overlap of 50 mm. For the 25-mm overlap, the results of the WC joint fall within the standard deviation of the DW joints. Samples containing cork agglomerate benefited the most from the overlap length increase and presented a safe and predictable failure mechanism.

### 3.3. Effect of the Testing Rate on Failure Load and Energy Absorption

To study the effect of different cross-head rates on the behaviour of the joints, W, DW, and WC joints were manufactured with an overlap length of 50 mm and tested in three conditions: quasi-static (0.001 m/min), high cross-head rate (6 m/min), and impact (60 m/min). An exception was considered for the set of DW samples subjected to impact conditions, where a rate of 120 m/min had to be applied to promote failure.

#### 3.3.1. Wood–Wood Joints (W)

**Figure 16:**
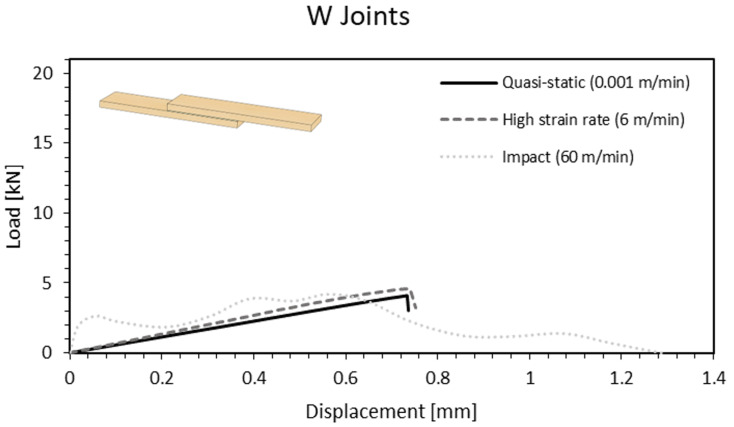
Load-displacement curve of a W joint as a function of the cross-head rate.

For this type of joint, the increase in stiffness and failure load was expected due to viscoelastic nature of polymers. The results showed an increase in terms of failure load of 12% and 40%, on average, for the high cross-head rate and the impact conditions, respectively (representative curves presented in Figure 16).

**Figure 17:**
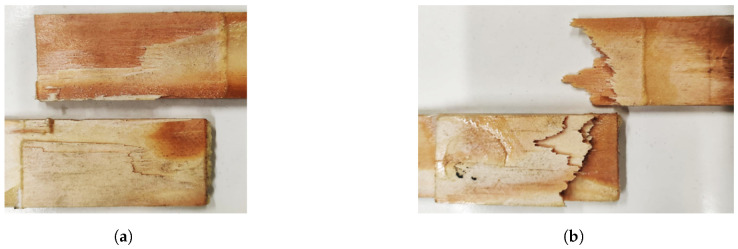

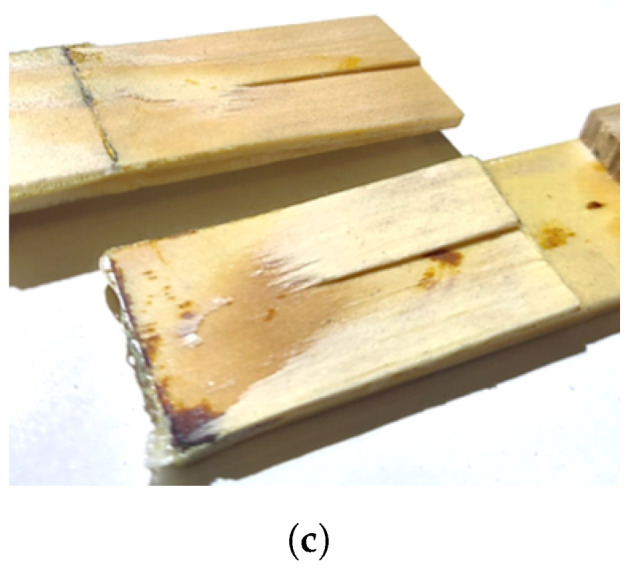
Failure mechanisms at different cross-head rates for W joints. (**a**,**b**) High cross-head rate (6 m/min). (**c**) Impact (60 m/min).

Regarding the failure modes, a combination of full substrate failure (Figure 17b) and severe delamination (Figure 17a,c) was observed. It was not possible to identify a clear trend for the failure mechanism of either the tests performed at high cross-head rate or under impact conditions. For the cases where delamination occured, penetration into the substrate was found to be higher than what was verified in quasi-static condition for a 25-mm overlap length.

#### 3.3.2. Densified Wood—Densified Wood Joints (DW)

**Figure 18:**
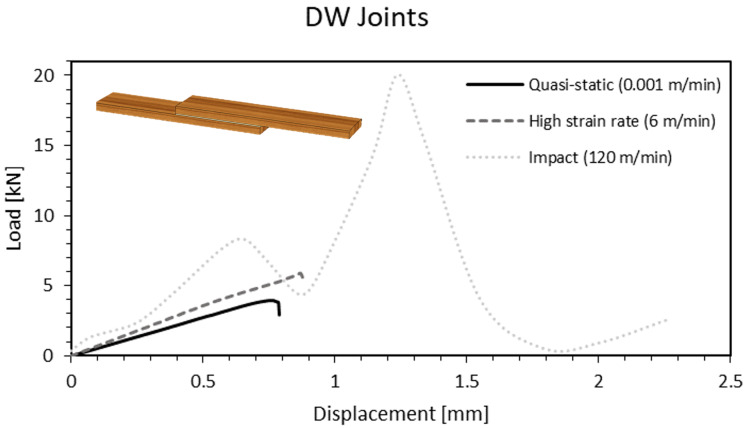
Load-displacement curve of a W joint as a function of the cross-head rate.

Densified timber joints behaved in a similar manner to natural pine when the cross-head rate was increased to 6 m/min, resulting in a 57% increase of the failure load when compared to quasi-static condition. However, under impact conditions at 60 m/min the specimens did not fail; thus, these were exceptionally tested at a rate of 120 m/min to promote failure. Surprisingly, such results led an average increase of 370% in the failure load, suggesting an extreme sensitivity of the joint to higher cross-head rates (Figure 18).

**Figure 19:**
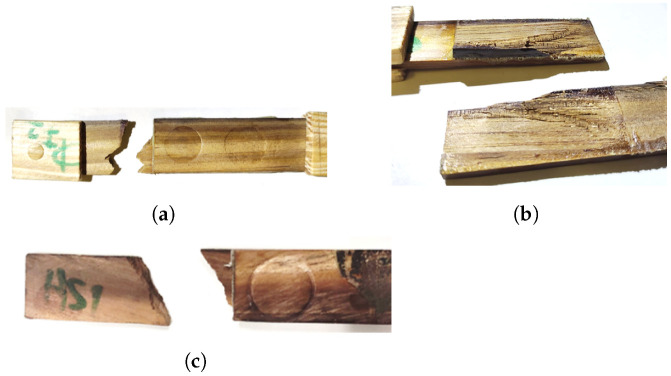
Failure mechanisms at different cross-head rates for DW joints. (**a**,**b**) Impact (120 m/min). (**c**) High cross-head rate (6 m/min).

The analysis of the fracture surfaces of the densified joints did not show any significant difference that could explain the large increase in strength witnessed under impact conditions. As was the case for the W joints, both rates tested resulted in severe delamination (Figure 19a) or failure on the substrate more or less close to the grips (Figure 19b,c). Thus, there is an indication that the wood substrates are responding in a significantly viscoelastic manner, providing additional strength as the testing rate is increased.

#### 3.3.3. Wood/Cork–Cork/Wood Joints (WC)

**Figure 20:**
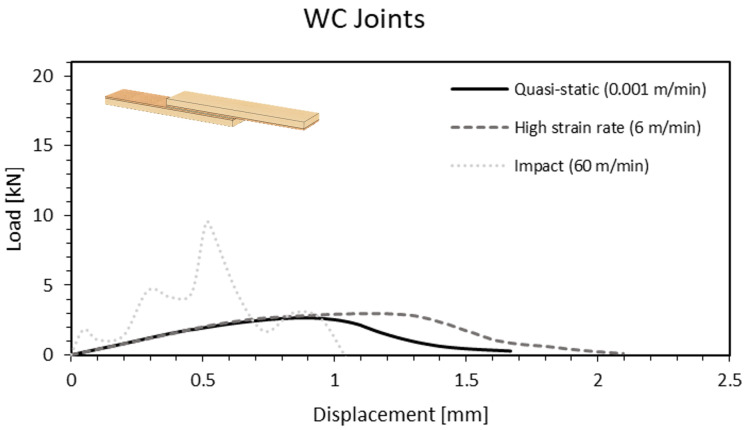
Load-displacement curve of a WC joint as a function of the cross-head rate.

Cork-coated joints revealed a slight 8.5% increase in displacement at failure when the cross-head rate was 6 m/min. This improvement rose to 125% when referring to impact conditions, on average (Figure 20).

**Figure 21:**
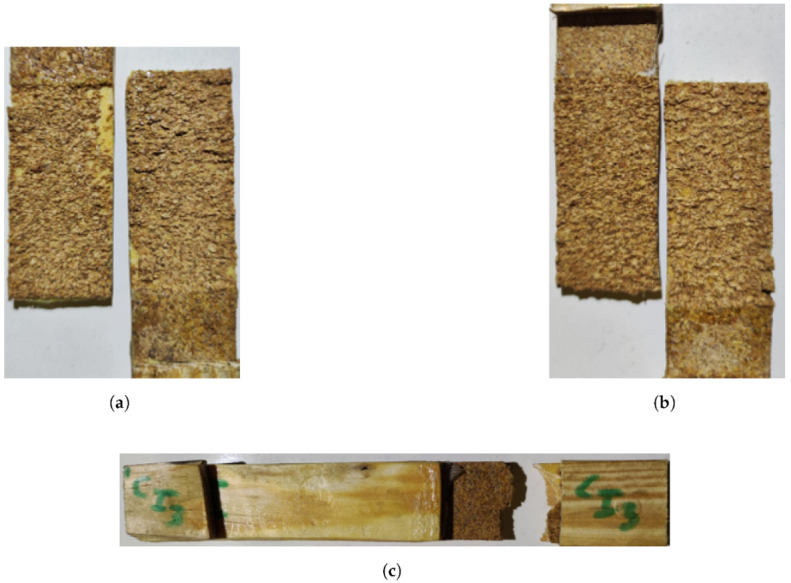
Failure mechanisms at different cross-head rates for WC joints. (**a**) High cross-head rate (6 m/min). (**b**) Impact; cohesive failure in the cork (60 m/min). (**c**) Impact; substrate failure (60 m/min).

The fracture surfaces revealed, for the majority of the tests, a safe and gradual failure mode already experienced in quasi-static conditions, where the behaviour of the joint was defined by the cork-adhesive phase (Figure 21a,b). Nevertheless, one result from impact conditions stood out due to a switch to a failure in the timber substrate (Figure 21c). This might indicate that a limit is being reached and there is a scenario of an increase in the cross-head rate or, eventually, the overlap length of the failure load would be limited by the strength of the timber.

#### 3.3.4. Comparison between Different Testing Rates

**Figure 22:**
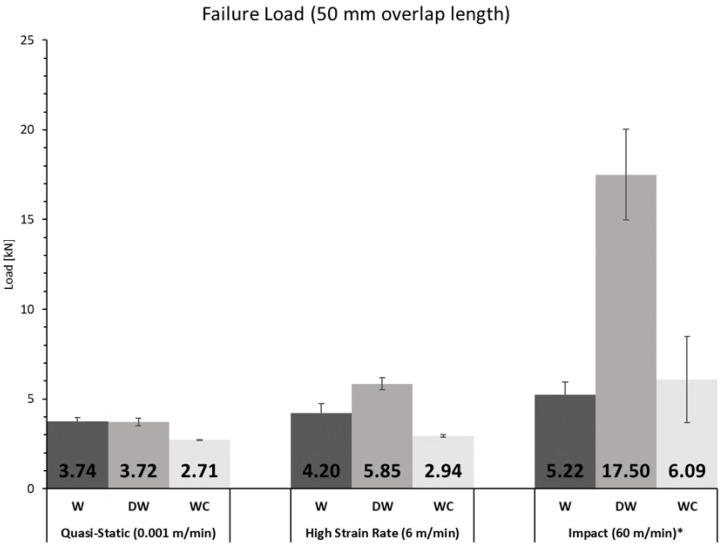
Summary of the failure load for each configuration and cross-head rate (* For DW 120 m/min are considered since the joint did not break for 60 m/min).

**Figure 23:**
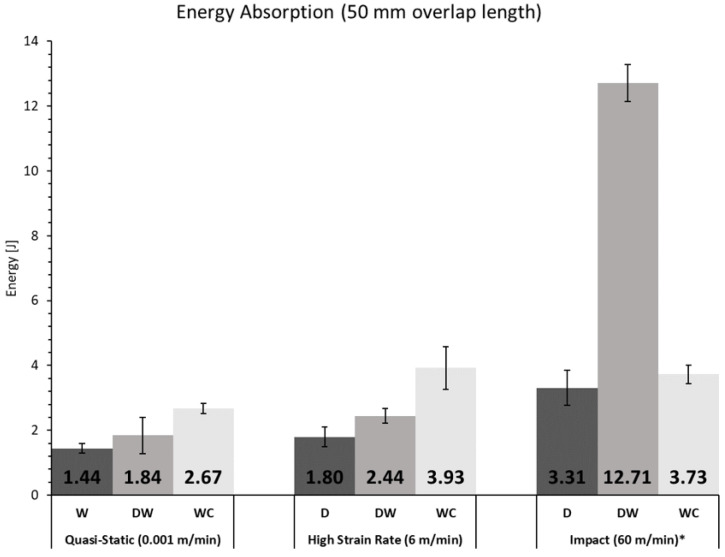
Summary of the energy absorption values for each configuration and cross-head rate (* For DW 120 m/min are considered since the joint did not break for 60 m/min).

In Figure 22 and Figure 23, a summary of the results in terms of failure load and energy absorption for each testing condition is displayed.

It can be seen that for the overlap length of 50 mm, for W, DW, or WC joints, the failure load increased with increasing testing rate, which can be attributed to the viscoelasticity of the materials used. For the quasi-static and high strain rate tests, although the failure load of the joints with WC joints was lower than that of the W and DW joints, the energy absorbed by these joints was higher. For impact the same was not verified because failure was not on the cork agglomerate, which happened for the other two test speeds, but in the wood substrate, not taking advantage of the cork energy absorption. For the two higher testing speeds, the failure load and energy absorbed by the joint increased from the W joints to the DW joints, taking advantage of the increase in strength of the densified wood. However, the same is not true for the quasi-static speed, because failure was not in the substrate but it moved to the adhesive.

## 4. Numerical Analysis

### 4.1. General Considerations

**Figure 24:**
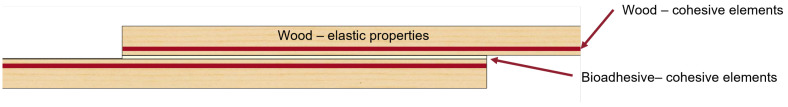
Illustration of the numerical model.

Aiming to accurately reproduce the behaviour of some of the SLJs tested experimentally, a static numerical model was developed for W joints and a 25-mm overlap length. The adhesive layer was modelled with cohesive elements. The behaviour of these elements was dictated by a triangular cohesive law that was defined via strength and fracture properties partially determined experimentally. Wood substrates were modelled as orthotropic with elastic properties obtained from Table 1. Furthermore, a layer of cohesive elements was placed within 0.2 mm of the interface to allow potential delamination to occur. These elements employed a triangular cohesive law, defined by strength and fracture properties of natural pine wood determined experimentally by Moura and Dourado [27]. A scheme of the model is presented in Figure 24.

Regarding the definition of a triangular cohesive law to model damage in timber, experimental results from Moura and Dourado [27] were used and included not only elastic and strength properties (Table 1 and Table 2) but also the required fracture properties such as $G_{Ic}$ and $G_{IIc}$. For modelling the bio-adhesive’s cohesive law, $G_{Ic}$ and $G_{IIc}$ values mentioned in Section 2 were used. Elastic and strength properties were only estimated, based on the on the brittle behaviour of the adhesive and considering a minimum strength in mode II of 12.40 MPa as seen in Section 2. A quadratic nominal stress criterion was used to define damage initiation. State variable SDEG was requested to track the damage evolution in the cohesive layers, given by fracture properties inputted. Because no strain rate-dependent data is available, modelling was only carried out for quasi-static conditions.

### 4.2. Boundary Conditions

**Figure 25:**
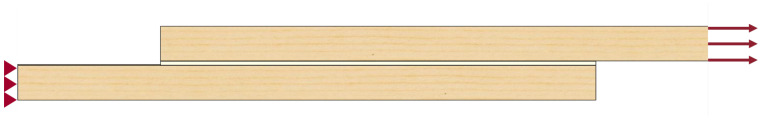
Illustration of the boundary conditions applied.

The boundary conditions were set by defining one of the edges as pinned and applying a displacement in the opposite edge, reproducing the experimental testing conditons, as shown in Figure 25.

### 4.3. Mesh

**Figure 26:**
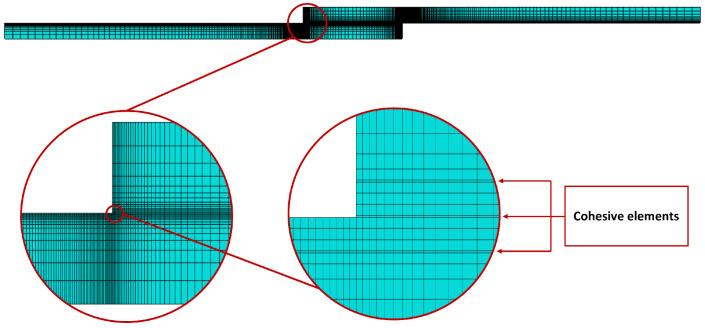
Mesh used in the simulations.

The mesh utilised for the simulation was refined, and the elements were distributed with a bias toward the areas where stress concentrations are higher, as observed in Figure 26. CPS4R plane stress elements were used for the elastic area of wood and COH2D4 cohesive elements were used for the cohesive layers.

#### Numerical Results

**Figure 27:**
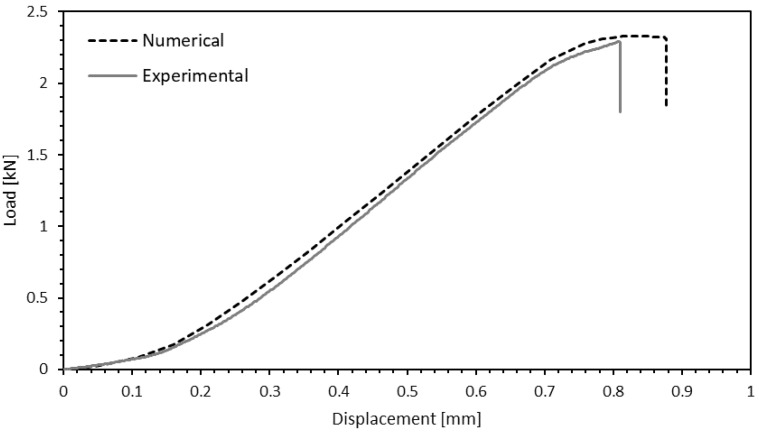
Comparison between numerical and experimental load-displacement curves.

Results show a general agreement with experimental data, as visualised in Figure 27.

**Figure 28:**
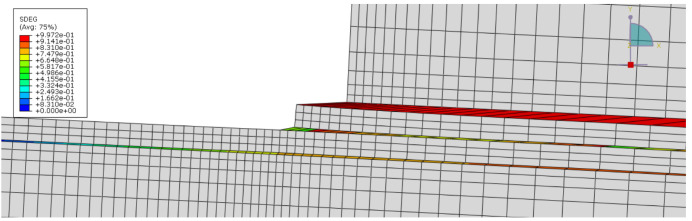
Simulated failure mechanism.

Regarding failure mode, the model also was found to be accurate. Figure 28 shows that prior to complete separation of the joint, cohesive elements of both the adhesive layer and timber are already heavily damaged, indicating a mixed failure of wood and adhesive. Therefore, the result is somewhat similar to what was observed experimentally, where partial delamination occurred most frequently.

## 5. Conclusions

In this work, wood/cork composites and densified pine wood were proposed as substrates and used to manufacture SLJs bonded with a novel polyurethane-based bio-adhesive. The performance of these joints was compared against that of joints with natural wood substrates.

The densification process was developed and successfully optimised to produce consistent batches of densified wood with an increase in density of 123 ± 16%, through the implementation of a mould and the adjustment of the treatment conditions.

Joint testing showed that the increase from 25- to 50-mm overlap length had the greatest effect on the wood/cork samples, which were found to have performed better in terms of energy absorption, because the larger overlap provides a more favorable stress field. The increase in energy was, for 50 mm of overlap, 85% when compared to the joints with wood substrates and 45% when compared to the joints with densified wood substrates. Therefore, in applications that require high-energy absorption, such as vehicles, cork can introduce interesting properties to the joint.

Only the wood/cork samples were able to result in a consistent failure mode, while the increase of the overlap length promoted wood failure for the wood and densified wood samples.

The lack of flatness of the wood surface resulted in poor bonding in some densified samples because the adhesive requires zero thickness bondlines.

Failure load and energy absorption were positively affected by strain rate due to viscolasticity of the materials. From the quasi-static testing rate to the impact rate, the energy absorbed increased 115%, 591%, and 40% for joints with wood, densified wood, and wood/cork substrates.

The numerical models developed exhibited a good correlation to the experimental results obtained.

## Figures and Tables

**Table 1:** Nominal elastic properties of pine wood [27].

$E_{L}$ [GPa]	$E_{R}$ [GPa]	$E_{T}$ [GPa]	$\nu_{\mathrm{LT}}$	$\nu_{\mathrm{LR}}$	$\nu_{\mathrm{TR}}$	$G_{\mathrm{LR}}$ [GPa]	$G_{\mathrm{LT}}$ [GPa]	$G_{\mathrm{TR}}$ [GPa]
12.0	1.91	1.01	0.51	0.47	0.31	1.12	1.04	0.29

**Table 2:** Nominal strength properties of pine wood [27].

$\sigma_{L}$ [MPa]	$\sigma_{R}$ [MPa]	$\sigma_{T}$ [MPa]	$\sigma_{\mathrm{LR}}$ [MPa]	$\sigma_{\mathrm{LT}}$ [MPa]	$\sigma_{\mathrm{RT}}$ [MPa]
97.5	7.9	4.2	16.0	16.0	4.5

**Table 3:** Tested joint configurations, highlighting the overlap length (in mm) tested for each condition.

	Q-S (0.001 m/min)	HCR (6 m/min)	Impact (60 m/min)
**W**	25	50	50
	50		
**DW**	25	50	50
	50		
**WC**	25	50	50
	50		

**Table 4:** Influence of the use of a mould in the density increase.

	**Initial Density** **[g/** ${\mathrm{cm}}^{3}$ **]**	**Final Density** **[g/** ${\mathrm{cm}}^{3}$ **]**	**Increase**
**Without mould**	0.676	1.07	58%
	0.653	1.06	62%
	0.642	1.07	67%
**With mould**	0.628	1.17	86%
	0.612	1.32	115%
	0.570	1.21	113%
	0.564	1.25	123%

## Data Availability

Not applicable.
